# Regorafenib synergizes with TAS102 against multiple gastrointestinal cancers and overcomes cancer stemness, trifluridine-induced angiogenesis, ERK1/2 and STAT3 signaling regardless of KRAS or BRAF mutational status

**DOI:** 10.18632/oncotarget.28602

**Published:** 2024-07-02

**Authors:** Jun Zhang, Lanlan Zhou, Shuai Zhao, Wafik S. El-Deiry

**Affiliations:** ^1^Molecular Therapeutics Program, Fox Chase Cancer Center, Philadelphia, PA 19111, USA; ^2^Laboratory of Translational Oncology and Experimental Cancer Therapeutics, Warren Alpert Medical School, Brown University, RI 02912, USA; ^3^Department of Pathology and Laboratory Medicine, Warren Alpert Medical School, Brown University, RI 02912, USA; ^4^The Joint Program in Cancer Biology, Brown University and Lifespan Health System, RI 02912, USA; ^5^Cancer Center at Brown University, Warren Alpert Medical School, Brown University, RI 02912, USA; ^6^Hematology-Oncology Division, Department of Medicine, Lifespan Health System and Warren Alpert Medical School, Brown University, RI 02912, USA

**Keywords:** TAS102, regorafenib, ERK1/2, angiogenesis, microvessel density

## Abstract

Single-agent TAS102 (trifluridine/tipiracil) and regorafenib are FDA-approved treatments for metastatic colorectal cancer (mCRC). We previously reported that regorafenib combined with a fluoropyrimidine can delay disease progression in clinical case reports of multidrug-resistant mCRC patients. We hypothesized that the combination of TAS102 and regorafenib may be active in CRC and other gastrointestinal (GI) cancers and may in the future provide a treatment option for patients with advanced GI cancer. We investigated the therapeutic effect of TAS102 in combination with regorafenib in preclinical studies employing cell culture, colonosphere assays that enrich for cancer stem cells, and *in vivo*. TAS102 in combination with regorafenib has synergistic activity against multiple GI cancers *in vitro* including colorectal and gastric cancer, but not liver cancer cells. TAS102 inhibits colonosphere formation and this effect is potentiated by regorafenib. *In vivo* anti-tumor effects of TAS102 plus regorafenib appear to be due to anti-proliferative effects, necrosis and angiogenesis inhibition. Growth inhibition by TAS102 plus regorafenib occurs in xenografted tumors regardless of p53, KRAS or BRAF mutations, although more potent tumor suppression was observed with wild-type p53. Regorafenib significantly inhibits TAS102-induced angiogenesis and microvessel density in xenografted tumors, as well inhibits TAS102-induced ERK1/2 activation regardless of RAS or BRAF status *in vivo*. TAS102 plus regorafenib is a synergistic drug combination in preclinical models of GI cancer, with regorafenib suppressing TAS102-induced increase in microvessel density and p-ERK as contributing mechanisms. The TAS102 plus regorafenib drug combination may be further tested in gastric and other GI cancers.

## INTRODUCTION

TAS-102 is an oral formulation consisting of trifluridine (FTD) and thymidine phosphorylase inhibitor tipiracil hydrochloride (TPI) at a molar ratio of 2:1, and is approved by the US FDA for the treatment of refractory metastatic colorectal cancer (rmCRC) and metastatic gastric cancer [1–4]. TAS102 monotherapy overcomes metastatic colorectal and gastric cancer progression failure with previous therapies [2, 4–6]. Recent studies assessed the efficacy of TAS-102 with the novel therapies including bevacizumab [7], panitumumab [8], nintedanib [9] and anti-PD-1 [10]. These studies indicated that TAS-102 in combination with diverse therapies can lead to survival benefits and restrict tumor progression. Regorafenib, a multi-target tyrosine kinase inhibitor, inhibits the activities of VEGFR2 and 3, Ret, Kit, PDGFR and Raf kinases and results in the inhibition of tumor angiogenesis and cell proliferation. Regorafenib improves the overall survival (OS) or progression free survival (PFS) in patients with gastrointestinal (GI) cancers after failure of standard treatments [2, 11–13]. Strategies combining regorafenib with diverse chemotherapies or immunotherapies have been observed to improve outcomes in multi-malignancies [14–17]. We previously reported that regorafenib in combination with fluoropyrimidine overcomes the progression in the refractory mCRC patients after failure of multiple 5-FU-containing combination therapies or regorafenib monotherapy [18]. Thus, we hypothesized that the novel strategy of TAS102 in combination with regorafenib could be investigated as an alternative regimen in the treatment of metastatic gastrointestinal cancers.

Cancer stem cells (CSCs) are a potentially crucial subpopulation of cancer cells that determine tumor initiation, self-renewal and heterogeneity. As a result, they contribute to tumor growth, recurrence, metastasis and chemo- and radio-resistance [19]. Targeting CSCs may provide a potentially curative approach to overcome therapy-resistance and prevent tumor progression [20]. Polymorphism analysis of MATE1/OCT2 predicts the efficacy and toxicity of TAS-102 in patients with refractory metastatic colorectal cancer [21]. Regorafenib suppresses acute myeloid leukemia (AML) engraftment *in vivo* and sensitizes CD34+ AML cells in the peripheral blood and spleen [22]. In addition, regorafenib disrupts the tumor-promoting interaction between tumor cells and mesenchymal stem cells and inhibits the growth and metastasis of colon cancer [23]. Thus, we investigated the combinatorial effect of TAS102 plus regorafenib on cancer stem cells. The over-growth and abnormal energetic metabolism usually lead to tumor necrosis, which is closely associated with high clinical stages and poor prognosis [24]. Moreover, necrosis modulates tumor angiogenesis, proliferation and invasion and promotes tumor progression and therapy-resistance [25, 26]. Regorafenib functions as a anti-angiogenesis receptor tyrosine kinase inhibitor for cancer therapy. Thus, we assessed the impact of regorafenib and TAS102 on tumor necrosis and angiogenesis.

In this study, we investigated the therapeutic effects and the underlying mechanisms of TAS-102 in combination with regorafenib against gastrointestinal cancers. The assessment of combination index showed that TAS102 synergizes with regorafenib in multiple GI cancers. TAS-102 in combination with regorafenib inhibits the formation of colonospheres, reduces the CD133+ subpopulation, as well the expression of ALDH1a. Experiments in mouse models showed that either TAS102 or regorafenib monotherapy can significantly ameliorate tumor burden *in vivo*. Moreover, TAS102 synergy with regorafenib alleviates tumor burden in a p53-dependent manner. Regorafenib significantly attenuates the microvessel formation in xenografted tumor tissues. Moreover, regorafenib abrogates the angiogenic promotion of TAS102 in CRC cells and xenografted tumor tissues harboring with BRAF V600E mutation. In addition, regorafenib modulates STAT3 and thymidylate synthase (TS) signaling.

## RESULTS

### TAS102 synergizes with regorafenib in multiple gastrointestinal cancer cell lines

**Figure 1:**
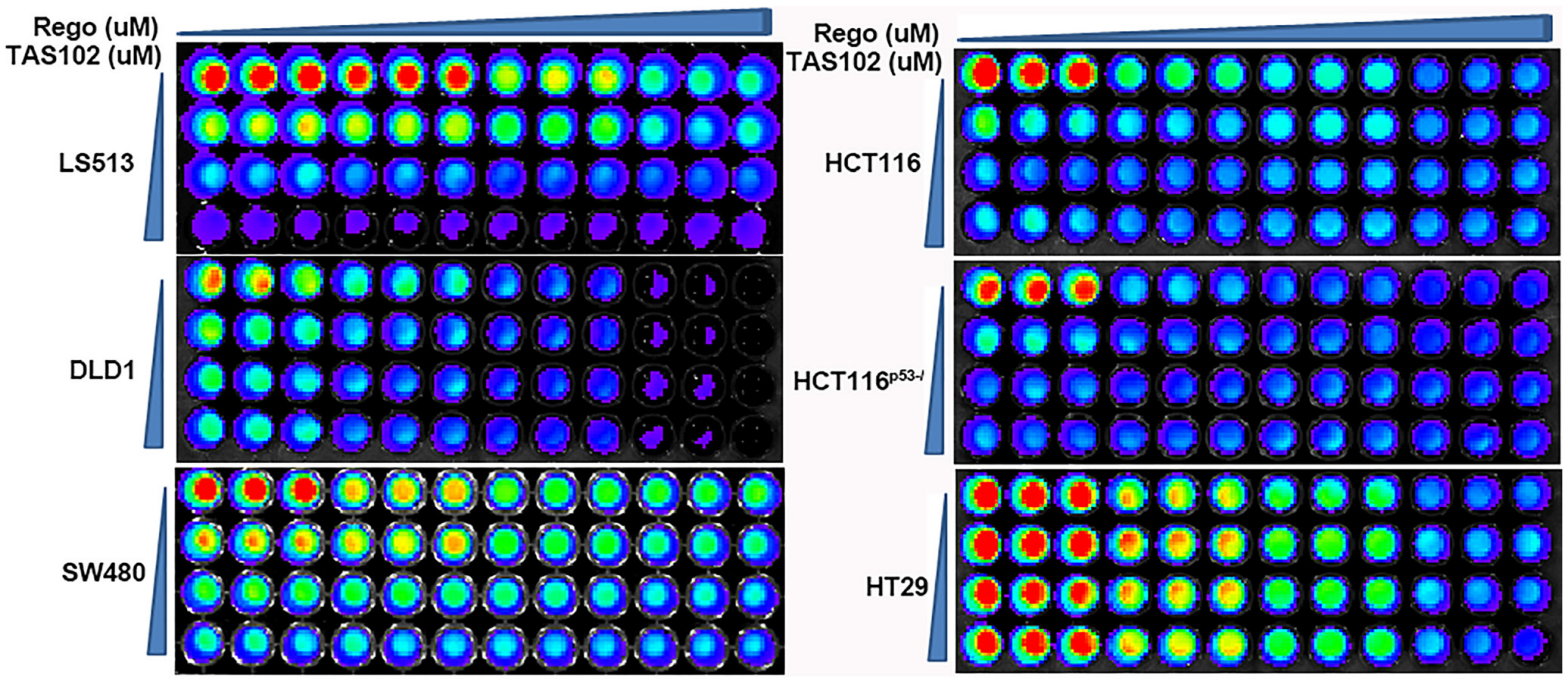
Synergy assessment of TAS102 plus regorafenib in multiple colorectal cancer cells. A panel of 6 CRC cells were treated with TAS102 in combination with regorafenib at these different doses for 48 hours. Representative fluorescent images of TAS102 in combination with regorafenib in the indicated CRC cell lines are shown.

A panel of seven human colorectal cancer cell lines were treated with varying concentrations of TAS-102 for 48 h (Table 1A). The results show that CRC cells with different driver gene subtypes are vulnerable to TAS-102 at a low dose (0.80 ± 0.57 µM of IC_50_ value), compared with normal cell lines (167.7 ± 28.4 µM of IC_50_ value) (Table 1A). We previously reported that fluoropyrimidine in combination with regorafenib may delay progression in mCRC patients after failure of multiple 5-FU-containing therapies and regorafenib monotherapy [18]. A recent study indicates that patients who were treated with FTD + TPI before switching to regorafenib had high adherence and persistence outcomes [27]. To investigate the combining effect of TAS102 plus regorafenib, we assessed the combination index of TAS102 with regorafenib *in vitro*. The results show that TAS102 can synergize with regorafenib in a variety of CRC cell lines with diverse driver gene subtypes at the indicated doses (Figure 1 and Table 1B). We further assessed the combination index of TAS102 plus regorafenib in gastric, pancreatic and liver cancer cells. The results show that TAS102 can synergize or superimpose with regorafenib in pancreatic and gastric cancer cells at the indicated doses (Table 1C). There was no significant synergy of TAS102 with regorafenib in a panel of 4 liver cancer cell lines in this study (Table 1C). The combination indices of TAS102 plus regorafenib are approximately 1 in liver cancer cells, which indicates an effect in liver cancer cells (Table 1C).

**Table 1:** Synergy assessment of TAS102 plus regorafenib in multiple gastrointestinal cancer cell lines

**Table 1A:** IC_50_ values of TAS102 in CRC cell lines with diverse gene subtypes

Cell lines	p53 status	KRAS status	BRAF status	IC_50_ (µM)	Mean ± SD
HCT116	WT	G13D	WT	0.394	0.80 ± 0.57
HCT116^p53–/–^	–	G13D	WT	0.422	
DLD1	S241F	G13D	WT	0.813	
LS513	WT	G12D	WT	1.012	
SW480	R273H; P309S	G12V	WT	0.999	
RKO	WT	WT	V600E	0.104	
HT29	R273H	WT	V600E	1.83	
MRC5	WT	–	–	147.6	167.7 ± 28.4
Wi38	WT	–	–	187.8	

Tables of half-maximal inhibitory concentration (IC_50_) in a panel of 7 human colon cancer cell lines and 2 normal cell lines after TAS102 treatment for 48 hours with a diverse concentration gradient.

**Table 1B:** The combination index (CI) of TAS102 plus regorafenib in CRC cells

TAS102 (µM)	Rego (µM)	CIs of CRC cells					
		LS513	DLD1	HCT-116	HCT116^p53–/–^	HT-29	SW-480
0.1/0.5	0.5/1	**0.52 ± 0.02**	**0.75 ± 0.07**	**0.75 ± 0.14**	**0.85 ± 0.07**	1.43 ± 0.16	1.96 ± 0.21
0.1/0.5	1/2	**0.85 ± 0.01**	**0.86 ± 0.07**	1.80 ± 0.22	1.27 ± 0.21	1.20 ± 0.04	**0.94 ± 0.09**
0.1/0.5	2/4	1.80 ± 0.19	**0.69 ± 0.13**	1.42 ± 0.14	1.14 ± 0.07	**0.93 ± 0.04**	**0.94 ± 0.01**
0.2/1	0.5/1	**0.43 ± 0.07**	**0.91 ± 0.06**	**0.50 ± 0.03**	**0.62 ± 0.07**	1.68 ± 0.03	**0.92 ± 0.05**
0.2/1	1/2	**0.63 ± 0.03**	1.00 ± 0.03	1.22 ± 0.19	1.57 ± 0.03	1.25 ± 0.05	**0.89 ± 0.07**
0.2/1	2/4	1.52 ± 0.21	**0.89 ± 0.17**	1.45 ± 0.04	1.89 ± 0.05	**0.89 ± 0.01**	1.00 ± 0.08
0.4/2	0.5/1	**0.74 ± 0.05**	1.34 ± 0.05	**0.97 ± 0.07**	1.34 ± 0.13	2.63 ± 0.14	1.06 ± 0.07
0.4/2	1/2	**0.96 ± 0.03**	1.35 ± 0.09	2.08 ± 0.03	2.14 ± 0.12	1.74 ± 0.05	**0.86 ± 0.01**
0.4/2	2/4	2.14 ± 0.25	1.03 ± 0.15	2.06 ± 0.02	2.16 ± 0.26	1.12 ± 0.15	1.07 ± 0.06

Table of combination indices (CIs) in a panel of 6 CRC cell lines treated by TAS102 in combination with regorafenib for 48 hours at the indicated doses.

**Table 1C:** The combination index of TAS102 plus regorafenib in gastrointestinal (GI) cancer cells

TAS102 (µM)	Rego (µM)	CIs of GI cancer cells							
		Gastric cancer cells		Pancreatic cancer cells		Liver cancer cells			
		AGS	SGC7901	Mia-paca-2	PANC1	HepG2	Huh7	PLC/PRF/5	Sk-Hep1
1	1/2	**0.91 ± 0.04**	1.08 ± 0.04	1.11 ± 0.03	1.12 ± 0.20	1.75 ± 0.15	1.17 ± 0.10	1.70 ± 0.33	1.27 ± 0.21
1	2/4	**0.89 ± 0.07**	1.29 ± 0.02	1.19 ± 0.02	**0.85 ± 0.01**	1.51 ± 0.04	2.18 ± 0.25	1.46 ± 0.37	1.28 ± 0.12
1	4/8	**0.99 ± 0.07**	**0.88 ± 0.00**	1.28 ± 0.06	1.25 ± 0.00	1.16 ± 0.06	1.19 ± 0.04	0.97 ± 0.03	1.27 ± 0.11
2	1/2	**0.84 ± 0.02**	**0.86 ± 0.03**	1.00 ± 0.01	**0.64 ± 0.01**	1.43 ± 0.32	1.47 ± 0.38	1.45 ± 0.11	1.68 ± 0.44
2	2/4	**0.81 ± 0.08**	1.0 ± 0.02	1.11 ± 0.01	**0.81 ± 0.00**	1.28 ± 0.30	1.77 ± 0.14	2.10 ± 0.72	1.83 ± 0.28
2	4/8	**0.85 ± 0.05**	**0.81 ± 0.05**	1.10 ± 0.01	1.13 ± 0.06	1.21 ± 0.08	1.29 ± 0.11	1.41 ± 0.27	1.47 ± 0.08
4	1/2	**0.81 ± 0.01**	**0.84 ± 0.04**	**0.95 ± 0.01**	1.13 ± 0.03	1.20 ± 0.17	1.57 ± 0.26	1.12 ± 0.12	1.21 ± 0.07
4	2/4	**0.81 ± 0.05**	**0.83 ± 0.02**	**0.89 ± 0.01**	1.18 ± 0.03	1.22 ± 0.12	1.79 ± 0.09	1.35 ± 0.45	1.26 ± 0.11
4	4/8	**0.71 ± 0.03**	**0.68 ± 0.03**	**0.91 ± 0.01**	1.61 ± 0.03	1.18 ± 0.05	1.44 ± 0.07	1.41 ± 0.07	1.58 ± 0.01

Table of CIs in gastric, pancreatic and liver cancer cells treated by TAS102 in combination with regorafenib at the indicated doses. CI <1, =1 and >1 respectively indicate synergy, additive effects or antagonism. Bold numbers highlight synergy at the indicated doses.

### TAS102 in combination with regorafenib alleviates the stemness of CRC cells

**Figure 2:**
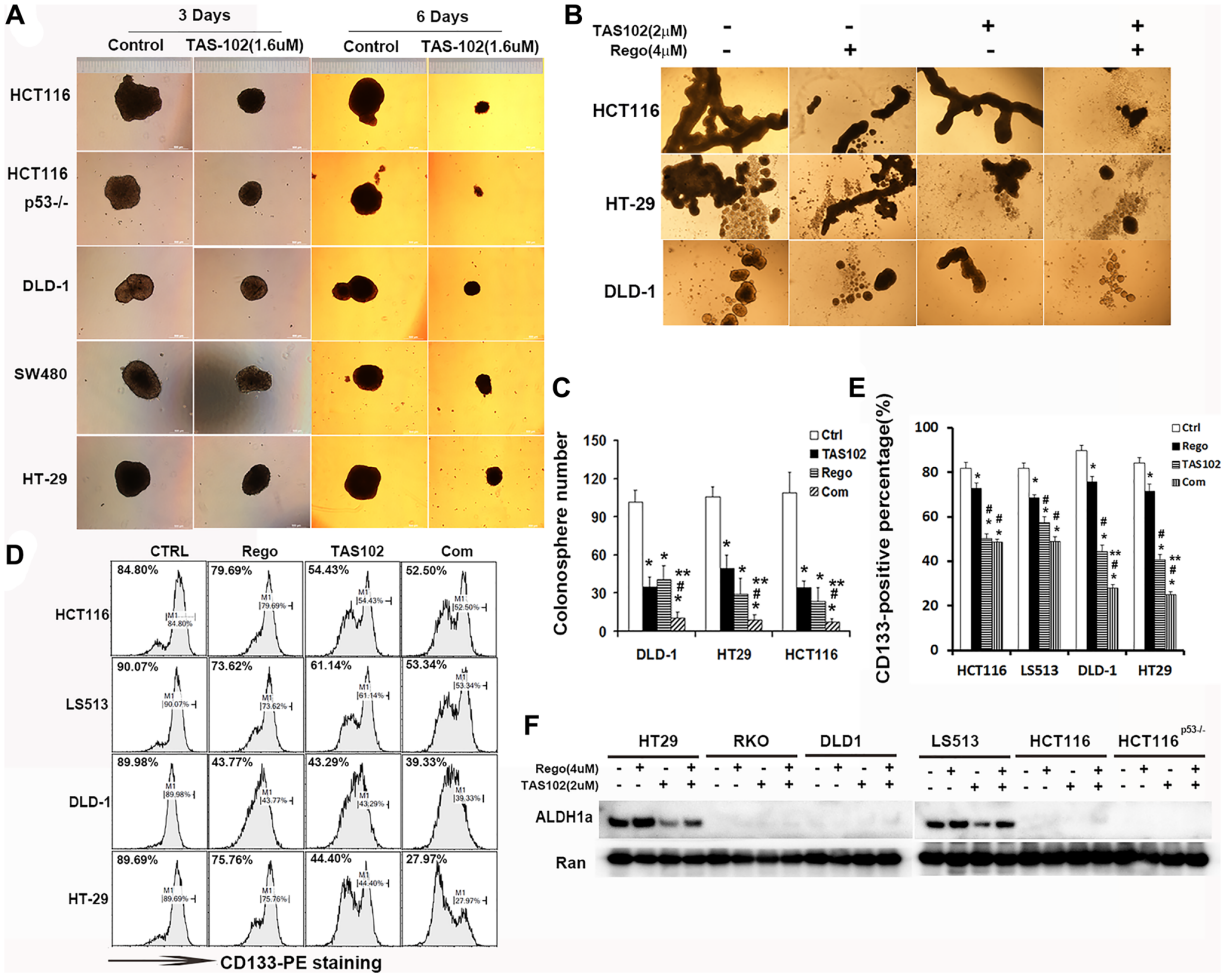
Combination of TAS102 (trifluridine/tipiracil) with regorafenib inhibits the stemness of colorectal cancer cells. (**A**) The effect of single-agent TAS102 on sphere formation with 5 colon cancer cell lines for up to 6 days. Representative images of the colonospheres are shown. (**B**, **C**) Formation of colonospheres after 2 μM TAS102 in combination with 4 μM regorafenib treatment for 6 days. (B) Representative images of the colonospheres are shown. (C) The number of colonosphere is calculated and statistically analyzed. The diagram shows the colonosphere number from these different CRC cells with the indicated treatment. (**D**, **E**) The CD133 positive cell population are assessed using flow cytometry after 2 μM TAS102 in combination with 4 μM regorafenib treatment for 48h. (D) Representative graphs are shown. (E) Bar diagrams represent CD133 positive percentage. (**F**) The expression of ALDH1a is assessed by western blotting after 2 μM TAS102 in combination with 4 μM regorafenib treatment for 48 h. Representative bands are shown. Data are shown as Mean ± SEM (Student’s *t*-test, 2-tailed, ^*^ *P* < 0.05 vs. Control, ^**^ *P* < 0.05 vs. TAS102 monotherapy, ^#^ *P* < 0.05 vs. regorafenib monotherapy). Each experiment was repeated at least 3 times.

Cancer stem-like cells (CSCs) contribute to a poor prognosis as well as resistance to chemotherapy in most malignancies [28]. The formation of floating spheroids grown from CSCs can promote tumor heterogeneity and resistance to chemotherapeutic agents [29, 30]. We assessed the effect of TAS102 on tumor colonosphere formation. The results show that TAS102 attenuates sphere formation from cultured colon cancer cell lines with various driver gene subtypes (Figure 2A). The co-administration of TAS102 and regorafenib significantly restricts the formation of colonospheres either in size (Figure 2B) or in number (Figure 2C), as compared with regorafenib or TAS102 monotherapy. we evaluated the CD133+ cell subpopulation in CRC cells, the results show TAS102 or Regorafenib monotherapy attenuates CD133+ subpopulation in CRC cells, the inhibitory effect of TAS102 upon CD133+ subpopulation is significant in HCT116, LS513 and HT29 cells, compared with regorafenib monotherapy. TAS102 plus regorafenib significantly inhibits CD133+ subpopulation in HT29 and LS513 cells (Figure 2D, 2E). Moreover, as ALDH1a is the common CSCs biomarker, we assessed the expression of ALDH1a in CRC cells. The results show that TAS102 administration can reduce the ALDH1a expression in HT29 and LS513 cells. Interestingly, regorafenib can induce the expression of ALDH1a in HT29 and LS513 cells, and TAS102 co-administration can reduce regorafenib-induced ALDH1a expression in HT29 cells (Figure 2F).

### TAS102 plus regorafenib reduces tumor growth in a p53-dependent manner

**Figure 3:**
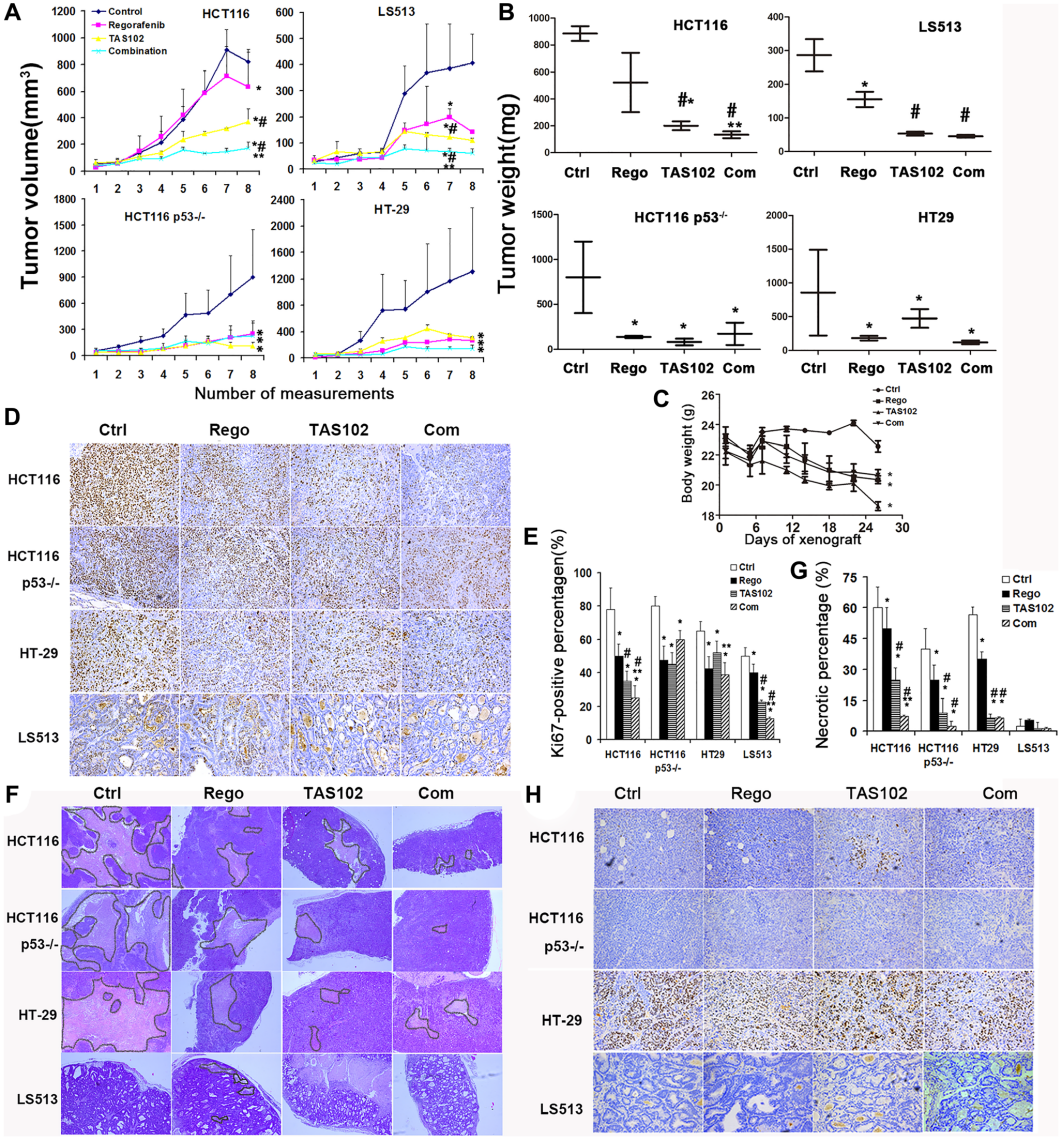
Therapeutic effects of TAS102 plus regorafenib in xenografted colorectal tumors. Nude mice with xenografted tumors were orally administered TAS102 100 mg/kg twice a day or/and 5 mg/kg regorafenib once per day for 2 weeks. (**A**) Xenografted tumor volume was measured at 2- or 3-day intervals for approximately 1 month and the growth curves are plotted. (**B**) Xenografted tumors were excised and weighed. (**C**) Body weight was measured at the indicated time-points. (**D**, **E**) Assessment of the proliferative percentage is shown using Ki-67 staining in xenografted tumors. (D) Representative images of Ki-67 staining in xenografted tumors are shown. Every image is magnified 100 times. (E) The diagram of Ki-67-positive percentage is shown and statistically analyzed. (**F**, **G**) Assessment of tumor necrosis in the xenografted tumors. (F) Representative images of hematoxylin-eosin (H&E) staining are shown. Every image is magnified 40 times. (G) The diagram of necrotic percentage is shown and statistically analyzed. (**H**) p53 status is assessed and verified in tumor tissues xenografted from the indicated CRC cells. Representative images of p53 staining in xenografted tumors are shown. Each image is magnified 100 times. Data are shown as Mean ± SEM (One-way ANOVA test, ^*^ *P* < 0.05 vs. Control, ^**^ *P* < 0.05 vs. TAS102 monotherapy, ^#^ *P* < 0.05 vs. regorafenib monotherapy).

We investigated the anti-tumor effects of TAS102 plus regorafenib in CRC-xenografted tumors. The data show that TAS102 or regorafenib monotherapy can significantly inhibit the growth of xenografted tumors with diverse driver gene mutations (Figure 3A, 3B). The growth inhibition of TAS102 in combination with regorafenib is increased in HCT116- and LS513-xenografted tumors harboring wild-type (WT) p53, as compared with TAS102 or regorafenib monotherapy (Figure 3A, 3B). The loss of body weight was approximately 10–15% after single-agent or co-administration of TAS102 and regorafenib for 3 weeks, as compared with the control (Figure 3C). The loss of body weight with co-administration of TAS102 and regorafenib is not significant, as compared with TAS102 or regorafenib monotherapy in this study (Figure 3C). IHC staining confirmed TP53 expression in these xenografted tumor tissues, the result shows that TAS102 can increase the focal expression of p53 in HCT116-grafted tumor tissues (Figure 3H). HT29-grafted tumors show an extensive expression as a mutant TP53 pattern (Figure 3H). Single-agent TAS102 or regorafenib significantly decrease the Ki-67 proliferative index in xenografted tumors (Figure 3D, 3E). The combination therapy with TAS102 and regorafenib significantly inhibits cell proliferation in HCT116- and LS513-xenografted tumors harboring wild-type p53, as compared with TAS102 or regorafenib monotherapy (Figure 3F, 3G). Auto-necrosis is a special characteristic of solid tumors. We found that single-agent TAS102 or regorafenib significantly inhibit tumor necrosis in these xenografted tumors. Single-agent TAS102 showed an enhanced inhibition of tumor necrosis, compared with single-agent regorafenib treatment (Figure 3F, 3G). In addition, TAS102 plus regorafenib significantly decreases HCT116-xenografted tumor necrosis, compared with single-agent treatment (Figure 3F, 3G). Interestingly, the necrosis is not obvious in LS513-xenografted tumors with or without the treatments of TAS102 or regorafenib (Figure 3F, 3G). Necrotic areas of tumors contribute to progression under hypoxic conditions, due to selection pressure and are particularly difficult to treat.

### Regorafenib abrogates tumor angiogenesis including TAS102-induced microvessel formation associated with BRAF V600E mutation *in vitro* and *in vivo*

**Figure 4:**
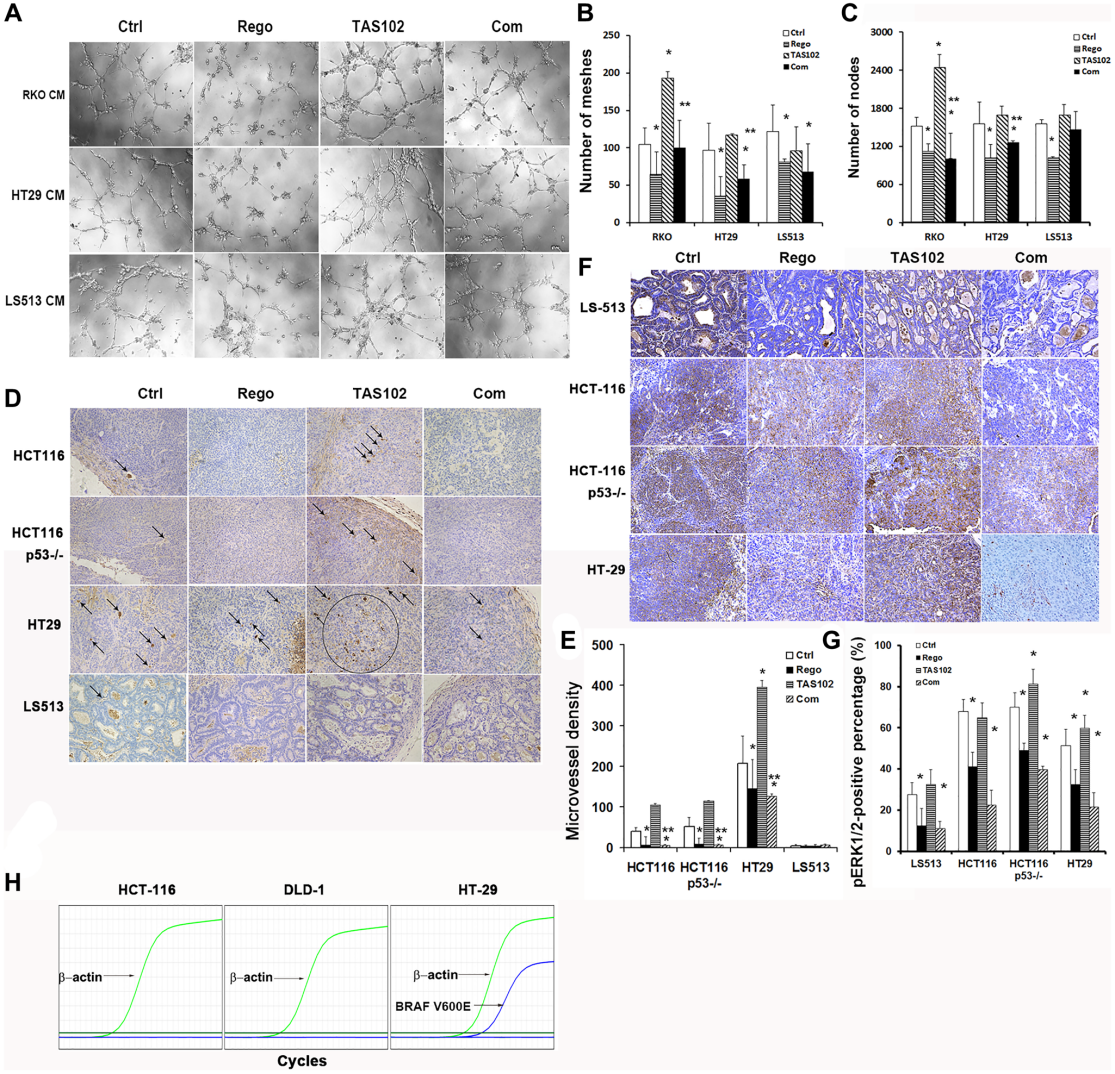
Tumor angiogenesis assessment of regorafenib in combination with TAS102 *in vitro* and *in vivo*. (**A**–**C**) RKO, HT29 and LS513 cells were cultured in 2 μM TAS102 and/or 4 μM regorafenib treatment for 48 h, and the conditioned media were collected to culture HUVEC endothelial cells for 6 h. Tube formation of HUVEC endothelial cells is photographed and analyzed using ImageJ Software plugin with angiogenesis analyzer. (A) Representative images of tube formation are shown. (B and C) The mesh number (B) and node number (C) of tube formation are statistically analyzed. (**D**–**G**) HCT116, HCT116^p53–/–^, LS513 and HT29 cells were xenografted in nude mice, TAS102 was orally administered at 100 mg/kg twice a day, regorafenib was orally administered at 5 mg/kg once a day. (D) Representative images of CD31 staining in the xenografted tumors are shown. The CD31-staining microvessels are labeled using black arrows in these images; more than 20 microvessels are circled in black. Each image is magnified 200 times. (E) The diagram of microvessel density is shown and statistically analyzed. Microvessels are counted using Image Pro Plus software. (F) Representative images of pERK1/2 (Thr202/Tyr204) IHC staining in xenografted tumors are shown. Every image is magnified 200 times. (G) pERK1/2-positive percentage in IHC slides is shown and statistically analyzed. (**H**) The status of BRAF is confirmed in HCT116, DLD1 and HT29 CRC cell lines using Quantitative PCR. The real-time PCR curves are shown. Data are shown as Mean ± SEM (One-way ANOVA test, ^*^ *P* < 0.05 vs. Control, ^**^ *P* < 0.05 vs. TAS102 monotherapy, ^#^ *P* < 0.05 vs. regorafenib monotherapy).

Tumor angiogenesis protects solid tumors from the necrosis induced by the over-growth and nutrient and oxygen deprivation, and thus contributes to tumor cell proliferation, tumor growth and metastasis of the viable tumor tissue [26]. We observed that the conditioned media (CM) containing regorafenib collected from different cultured CRC cells significantly inhibits HUVEC endothelial cell tube formation with or without TAS102 administration *in vitro* (Figure 4A), either in mesh number (Figure 4B) or node number (Figure 4C). In addition, HUVEC cell tube formation is increased in the conditioned media containing TAS102 monotherapy from cultured RKO and HT29 cells harboring BRAF mutation, whereas regorafenib co-administration can inhibit TAS102-induced tube formation *in vitro* (Figure 4A–4C). Furthermore, we investigated microvessel density in xenografted tumors with or without TAS102 and regorafenib treatment. The results show that regorafenib monotherapy inhibits the formation of microvessels in xenografted tumors with diverse driver gene mutations most likely through its anti-angiogenesis receptor tyrosine kinase inhibitory effects (Figure 4D, 4E). TAS102 monotherapy shows an obvious promotion of microvessel density in HT29-grafted tumors harboring with BRAF V600E mutation, whereas regorafenib co-administration inhibits TAS102-induced angiogenesis (Figure 4D, 4E). BRAF status was confirmed in various CRC cells, BRAF V600E mutation is detected in HT29 cells, unlike HCT116 and DLD1 cells (Figure 4H). The microvessels are rarely observed in LS513-xenografted tumors with or without TAS102 and regorafenib administration (Figure 4D, 4E). Prior studies indicated that blocking the ERK1/2-related pathway can inhibit angiogenesis in multiple malignancies [31–33]. As regorafenib functions as a RAF kinase inhibitor, we assessed the activity of ERK1/2/MAPK cascades *in vivo*. The results show that TAS102 monotherapy increases ERK1/2 activation in HT29- and HCT116^p53–/–^-xenografted tumor tissues (Figure 4F, 4G). By contrast, regorafenib administration significantly decreases phospho-ERK1/2 expression in tumor tissues, including TAS102-induced ERK1/2 activation (Figure 4F, 4G).

### TAS102 in combination with regorafenib inhibits ERK1/2 and STAT3 signaling

**Figure 5:**
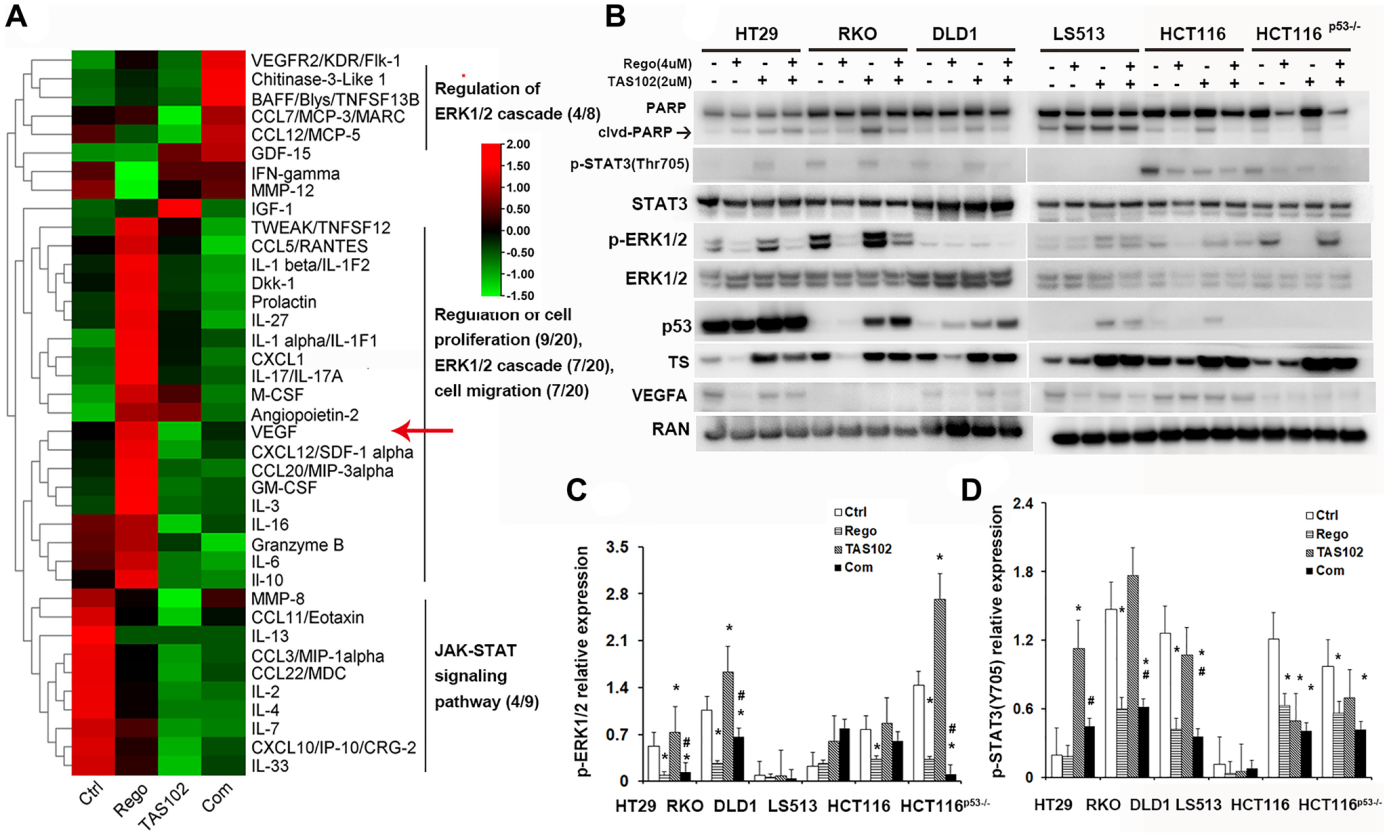
TAS102 in combination with regorafenib inhibits ERK1/2 and STAT3 signaling. (**A**) Nude mice with xenografted tumors were orally administered TAS102 100 mg/kg twice a day or/and 5 mg/kg regorafenib daily for 2 weeks. The mice were sacrificed and the plasma was collected. The relative content of cytokines in the plasma is depicted using heat map and analyzed using GO and KEGG enrichment analysis. (A) The heat map and the enriched pathways of cytokines with different treatments from GO and KEGG analysis are shown. (**B**) The indicated protein expression was evaluated using immunoblotting with or without therapy. (**C**, **D**) Average optical intensities (AOIs) of p-ERK1/2 and p-STAT3 are normalized to their total protein. RAN is used as a loading control. The AOIs of RAN or total protein is normalized as 1. Data are shown as Mean ± SEM (One-way ANOVA test, ^*^ *P* < 0.05 vs. Control, ^#^ *P* < 0.05 vs. TAS102 monotherapy).

We observed synergic effects of TAS102 in combination with regorafenib to reduce tumor growth, microvessel formation and tumor necrosis *in vitro* and *in vivo* (Figures 1–4). Furthermore, we analyzed cytokine profiles from the experimental mouse models with or without TAS102 or regorafenib treatment. The results suggest that the therapeutic mechanisms of TAS102 plus regorafenib involve regulation of the ERK1/2 pathway, cell proliferation, cell migration and the JAK-STAT3 pathway signaling (Figure 5A). Western blotting shows that regorafenib inhibits ERK1/2 and STAT3 activation in monotherapy and combination therapy, including overcoming TAS102 promotion of ERK1/2 activation in HT29, RKO and HCT116^p53–/–^ cells (Figure 5B, 5C), as well as TAS102-induced STAT3 activation in HT29, LS513, HCT116 and HCT116^p53–/–^ cells (Figure 5B, 5D). FTD is a fluorinated thymidine analogue and functions as a competitive inhibitor of thymidylate synthase (TS), as it can stimulate TS expression in CRC cells [34]. Upregulation of TS may contribute to therapeutic resistance to TAS102. Regorafenib functions as a multi-targeted tyrosine kinase inhibitor and its monotherapy reduces TS expression in HT29, RKO, DLD1 and HCT116 cells, and co-administration with regorafenib reduces TAS102-induced TS expression in HT29 cells (Figure 5B). Interestingly, regorafenib increases the content of VEGF in animal’s plasma (red arrow in Figure 5A), instead of the reduction observed in CRC cell lysates (Figure 5B).

## DISCUSSION

We provide evidence for synergy between two FDA-approved drugs to treat refractory metastatic gastrointestinal cancers using TAS102 and regorafenib. Our observations were made in cell culture, colonosphere assays, and *in vivo* xenografts of GI cancer mouse models. We observed an increase of pERK1/2 and microvessel density in TAS102-treated tumors harboring BRAF mutation and these effects were overcome when TAS102 was combined with regorafenib. We previously found synergy between regorafenib and 5-FU in preclinical models, and later observed delayed disease progression in case reports from Dr. El-Deiry’s clinic treating patients with advanced colorectal cancer. The current findings suggest that the combination of TAS102 and regorafenib has combinatorial efficacy in colorectal cancer and other GI cancers such as gastric or pancreatic cancer, and this should be further investigated in the clinic.

Combination of TAS102 with nintedanib, a novel triple angiokinase inhibitor, increases the DNA incorporation of trifluridine in xenografted tumors and is currently being investigated in the clinic [9]. TAS-102 in combination with bevacizumab, an inhibitor of vascular endothelial growth factor, exhibits a promising activity with manageable safety in the treatment of refractory mCRC patients (recently, the combination of TAS102 plus bevacizumab has been FDA approved during the publication process of this manuscript) [7]. Trifluridine/tripacil application or re-induction of chemotherapy and/or target therapy is effective following regorafenib therapy [27, 35].

We found that TAS102 can synergize with regorafenib in multiple gastrointestinal cancers including colon cancer, gastric and pancreatic cancer *in vitro* (Table 1 and Figure 1). Our *in vivo* studies show that TAS102 in combination with regorafenib significantly delays CRC-xenografted tumors harboring wild-type (WT) p53, as compared with TAS102 or regorafenib monotherapy (Figure 3). The loss of body weight treated by the co-administration of TAS102 and regorafenib is not significant, compared with TAS102 or regorafenib monotherapy (Figure 3C). Regorafenib exhibits both anti-proliferative and anti-angiogenetic activities as a multi-target tyrosine kinase inhibitor (TKI), thereby inhibiting growth and metastasis of colon cancer [23]. Trifluridine interferes with DNA synthesis and inhibits cell proliferation. Our data show that TAS102 in combination with regorafenib significantly inhibits tumor proliferation in xenografted tumors harboring WT p53 (Figure 3D, 3E). The population of cancer stem cells determines tumor self-renewal, recurrence, metastasis and drug-resistance. TAS102 in combination with regorafenib suppresses the formation of colonospheres and the CD133+ cancer stem cell sub-population in CRC cells (Figure 2). Tumor necrosis is a poor prognostic predictor and is positively associated with clinical staging in CRC patients [24]. Our data show the co-administration of TAS102 and regorafenib alleviates necrosis in HCT116-xenografted tumors (Figure 3F, 3G). Collectively, these findings suggest that TAS102 in combination with regorafenib may provide a novel effective strategy in treatment for patients with multiple GI cancers.

Therapeutic strategies targeting simultaneously drug-sensitive tumor cells and their cancer stem cells may have therapeutic benefits [36]. A recent study indicates that CD44/CD133-positive colorectal cancer stem cells are sensitive to trifluridine (FTD) exposure [37]. Regorafenib suppresses tumorigenesis and the generation of drug-resistant cancer stem-like cells via modulation of miR-34a associated signaling [38]. Our data show that trifluridine in combination with tipiracil (TPI) significantly restricts CRC stemness *in vitro* (Figure 2). Furthermore, TAS102 in combination with regorafenib inhibits growth of colonospheres and the CD133+ subpopulation (Figure 2B, 2E). In addition, the stemness marker ALDH1A1 promotes tumor angiogenesis via retinoic acid/HIF-1α/VEGF signaling in breast cancer cells [39]. Targeting S100A9-ALDH1A1-retinoic acid signaling can retract brain relapse in EGFR-mutant lung cancer [40]. Pharmaceutical inhibition of ALDH1a can re-sensitize ovarian cancer to platinum-based chemotherapy [41]. We observed that TAS102 can reduce regorafenib-induced ALDH1a expression in HT-29 cells (Figure 2F). Thus, these findings indicate that the synergistic combination mechanisms of TAS102 in combination with regorafenib simultaneously involve targeting CSCs in colon cancer. However, the underlying mechanism of TAS102 in combination with regorafenib targeting CSCs is complicated, and further studies are needed to explore more detailed mechanisms in the future.

p53 is a crucial mediator of the cell cycle checkpoint for growth arrest and DNA repair and p53 variant analysis can predict the efficacy of TAS-102 in mCRC patients [21, 42]. p53 status is an important predictor of HCC sensitivity to regorafenib therapy, as regorafenib significantly inhibits cell proliferation and promotes apoptosis in HCC cells harboring with wild-type p53 [43]. A prior study indicated that FTD induces arrest in the G2-phase of the cell cycle in a p53-dependent manner [34]. Our data demonstrate that TAS102 in combination with regorafenib shows an increasingly synergistic efficacy in restricting the growth and necrosis of xenografted tumors harboring wide-type p53, as compared with p53-null and p53 variant tumors (Figure 3A, 3B, 3D, 3E). Our results suggest that p53 status may in part indicate therapeutic efficacy of TAS102 in combination with regorafenib. Nonetheless, the combination therapy of TAS102 plus regorafenib appears to be active regardless of p53 status, KRAS or BRAF mutation.

Angiogenesis inhibition is a current therapy in the treatment of metastatic colorectal cancer via targeting tumor-driven angiogenesis [44]. Prior studies indicated that sorafenib, a multi-target tyrosine kinase inhibitor blocks the RAF/MEK/ERK pathway and inhibits the angiogenesis of hepatocellular carcinoma [31]. Angiotensin-converting enzyme 2 (ACE2) inhibits breast cancer angiogenesis via suppressing the VEGFa/VEGFR2/ERK pathway [32]. RhoJ (TC10-Like, TCL), a member of Rho GTPase Cdc42 subfamily, facilitates angiogenesis in glioblastoma via JNK/VEGFR2 mediated activation of PAK-BRAF-ERK signaling pathways [33]. Trifluridine can activate the ERK/AKT/STAT3 axis and induces pro-survival signaling in patient-derived CRC xenograft models [8]. We found that TAS102 monotherapy induces tumor angiogenesis in xenografted tumors with aberrant ERK/MAPK activation, whereas regorafenib co-administration abrogates the angiogenic promotion of TAS102 *in vitro* and in xenografted tumors (Figures 4 and 5B, 5C). Although the synergistic tumor growth inhibition by TAS102 in combination with regorafenib is not significant in HT29-xenografted tumors, the inhibition by regorafenib of TAS102-activated ERK and tumor angiogenesis supports the promising regimen of TAS102 plus regorafenib in CRC or other GI cancers with aberrant ERK/MAPK activation.

Overcoming FTD-activated ERK/AKT/STAT3 signaling provides a compelling rationale for testing and optimizing the combination strategy with TAS102 plus regorafenib against metastatic colorectal cancer [8]. As regorafenib exhibits a more potent inhibition to STAT3 than sorafenib, it can inhibit STAT3-related signaling to promote HCC cell apoptosis [45]. Regorafenib significantly increases the growth inhibition and apoptosis of HCC with aggressive CK19+ phenotype through inhibiting STAT3-induced mitochondrial respiratory enhancement [46]. We found that regorafenib administration restricts ERK1/2 and STAT3 activation in CRC cells and xenografted tumors with diverse gene subtypes, including TAS102-induced ERK1/2 or STAT3 activation (Figures 4F, 4G and 5B, 5D). Elevated thymidylate synthase (TS) induces CRC resistance to 5-FU therapy, and so inhibition of TS can sensitize CRC cell to 5-FU therapy [47]. We observed that regorafenib co-administration can deregulate TAS102-induced TS expression in HT29 cells. Collectively, these mechanistic findings support a novel combination regimen of TAS102 in combination with regorafenib against refractory colorectal or other GI cancers. We observe that regorafenib decreases VEGFA in CRC cell lysates and increases it in animal plasma. We assume that regorafenib inhibits the function of VEGFRs and reflexively stimulates the ligand secretion to maintain its function. However, further research is needed to unravel these relationships.

Although TAS102 (trifluridine/tipiracil) or regorafenib monotherapy is approved therapy in refractory mCRC, the studies involved in the combination are limited. Our findings including the *in vivo* anti-tumor effects and mechanistic insights involving regulation of tumor proliferation, necrosis and angiogenesis, as well as the predictors of drug sensitivity support a novel therapeutic rationale of TAS102 in combination with regorafenib against colon and other GI cancers. In particular, the pre-analysis of p53 and BRAF status is worth further testing in the clinical setting in correlative studies of TAS102 plus regorafenib. Further studies are warranted to investigate the effects of TAS102 on microvessel density and ERK1/2, and the mechanisms by which their effects can be suppressed by regorafenib. This may need to be done retrospectively as now TAS102 is approved in combination with bevacizumab.

Our preclinical studies suggest that the combination of FDA-approved drugs TAS102 and regorafenib are synergistic in cell culture, colonosphere assays and *in vivo*. The drug combination appears promising in colorectal cancer, gastric cancer, and pancreatic cancer, but not liver cancer. TAS102 appears to increase microvessel density and activate ERK1/2 in BRAF V600E-dependent manner, whereas these effects are suppressed with the combination with regorafenib. *In vivo* data in multiple models suggest the regimen is active regardless of KRAS, p53 or BRAF mutation although it appears more active when p53 is wild-type. Our findings provide the preclinical basis for an investigator-initiated trial in gastric, pancreatic, colon and other gastrointestinal cancers. Moreover, our results provide insights for biomarkers of drug efficacy such as microvessel density and phospho-ERK that may be evaluated in post-treatment biopsies.

## MATERIALS AND METHODS

### Reagents and cell lines

TAS102 and regorafenib were purchased from MedKoo Biosciences (Research Triangle Park, NC, USA), and were solubilized in DMSO at a storage concentration of 10 mM for cell experiments. Penicillin/Streptomycin, McCoy’s 5A, DMEM, EMEM, RPMI 1640, phosphate buffer saline, trypsin and fetal bovine serum (FBS) were purchased from GIBCO (Thermo Fisher Scientific, USA). The human colon cancer cell lines RKO, HT-29, DLD-1, SW-480 and LS513, gastric cancer cell lines AGS and SGC7901, pancreatic cancer cell lines Mia-paca-2 and Panc1, liver cancer cell lines HepG2, Huh7, PLC/PRF/5 and SK-Hep1, as well the human normal cell lines MRC-5 and Wi-38 were purchased from the American Type Culture Collection (ATCC, Manassas, VA, USA). HCT-116 and HCT-116 p53^–/–^ were generously provided by Dr. Bert Vogelstein (Johns Hopkins University, Baltimore, MD, USA). All cell lines were cultured in their ATCC-recommended media supplemented with 10–20% (v/v) FBS with or without chemotherapy agents at 37°C within a 95% humidified atmosphere containing 5% carbon dioxide in an incubator.

### Cell viability assays, colonosphere culture, CD133-positive cells subpopulation assessment

Cells were seeded in 96-well dark plates at a density of 2000–6000 cells per well and incubated overnight to allow proper attachment, the media were replaced using media with or without therapeutic agents for 48 hours. Subsequently cell viability was evaluated using a CellTiterGlo bioluminescence reagent (Promega Corporation, Madison, WI, USA), according to the manufacturer’s instructions.

Colonospheres were cultured using MammoCult Human Medium Kit (STEMCELL Technologies) plus heparin sodium (Merck, 4 mg/ml) and hydrocortisone (Merck, 0.5 mg/ml). The complete MammoCult medium was prepared for each experiment according to the manufacturer’s instructions. CRC cells were seeded in 24-well ultra-low attachment plates (Corning) at a density of 20,000 cells per well and incubated in the complete MammoCult media with or without treatment for 3–6 days. Colonospheres (>60 mm) were counted and imaged by light microscopy.

CRC cells were cultured in serum-free medium with diverse treatments for 48 h, and then cells were collected and stained using PE Anti-Human CD133 Antibody ((Elabscience Biotechnology, Wuhan, China), according to the manufacturer’s instruction. The stained cells were subjected to flow cytometric analysis to count CD133-positive cells and the percentage was calculated.

### Endothelial cell tube formation assay for *in vitro* study of angiogenesis

The HUVEC human endothelial cell line was purchased from the American Type Culture Collection (ATCC, Manassas, VA, USA) and cultured in the recommended medium. The conditioned media were collected from the solution that cultured CRC cells treated with TAS102 and Regorafenib for 48 h. A total of 15,000 HUVEC cells per well were seeded in 96-well plates coated with the solidified bottom matrigel formed by reduced growth factor basement membrane extracts (BME) (Corning 354230), cultured in the conditioned media for 6 h and photographed by microscopy. ImageJ software installed angiogenesis analyzer plugin was used to analyze and count these formed meshes and juncture nodes [48].

### Immunohistochemical (IHC) analysis and measurement of microvessel density

IHC was used to evaluate the expression of phospho-ERK1/2 (Thr202/Tyr204) (Cell Signaling Technology, Beverly, MA), CD31, TP53 and Ki-67 (ZSGB-BIO Inc. Beijing, China) in tumor tissues. The primary antibodies were detected by PV-9000 system-HRP-DAB kit (ZSGB-BIO Inc. Beijing) using Immuno-Histo Stainer (Leica Bond Max), according to the manufacturers’ instructions. IHC slides were observed and photographed using a luminescence microscope (Leica DMLB). The brown particles observed in cytoplasm (CD31 and pERK1/2 staining) or in nucleus (p53 and Ki-67 staining) were interpreted as positive cells in IHC slides. Microvessels were detected by CD31-IHC staining. Microvessel density was measured as previously described [49] with some modification and quantitatively analyzed using Image Pro Plus software.

### Necrosis assessment

Tumor tissues were fixed in 4% neural-buffered formalin and embedded in paraffin. The blocks were sectioned into 2–3 µm slides and subjected to H&E staining. Necrosis including intraluminal necrosis in H&E-staining slides was assessed as the previously described [24] and quantitatively analyzed using Image Pro Plus software.

### Dose-response assessment and synergy assessment

Half-maximal inhibitory concentration (IC_50_) value is a critical index of the cell viability dose-response curve. In this study, IC_50_ was used to assess single drug inhibition in cell viability experiments after treatment of 48 hours *in vitro*. Prism statistical software (GraphPad Software, La Jolla, CA) was used to calculate the IC_50_ values and to plot dose-response curves. The combinatorial inhibitory effects of multiple drugs were assessed using a quantitative analysis of dose-response relationships as described previously [50]. Combined effects of multiple doses were assessed by combination index (CI). Compusyn software was used to quantitatively analyze CI values based on Chou-Talalay analysis. If the sum of the 2 fractional terms is equal to 1, an additive effect is indicated. If the CI value is smaller than 1, synergy is indicated, and if the CI value is greater than 1, antagonism is indicated [51].

### *In vivo* tumor xenografts, cytokine profiling and drug administration

All animal experiments were conducted in accordance with IACUC regulations. 6-week-old female athymic nu/nu mice (Taconic Biosciences) were inoculated with 2 × 10^6^ cells of HCT116, HCT116 p53^−/−^, HT-29 and LS513 cell lines in a 200 ml suspension of Matrigel (BD) with PBS at a volume ratio of 1:1. All subcutaneous tumors were allowed to establish growing masses for 5 days after injection until they reached a volume of approximately 100–150 mm^3^ before treatment initiation. Tumor volume was measured with a caliper at 2- or 3-day intervals and calculated using the formula (length × width^2^)/2. Mice were sacrificed approximately 1 month later and tumors were excised and weighed.

Animal plasma was collected and subjected to cytokine analysis using 44-plex murine Luminex panel assays (R&D LXSAHM) on a Luminex 200 Instrument (R&D LX200-XPON-RUO), according to the manufacturer’s instruction. These data were depicted by heat map using RStudio software and analyzed using GO and KEEG enrichment analysis online (DAVID Functional Annotation Bioinformatics Microarray Analysis (ncifcrf.gov)).

TAS102 was dissolved in 0.5% hydroxypropyl methyl cellulose (HPMC, W/V) at a final concentration of 20 mg/ml [8]. Regorafenib was dissolved in a solution of polypropylene glycol/PEG400/poloxamer 188 (42.5%/42.5%/15%, W/V) at a final concentration of 1 mg/ml. TAS102 was orally administered at 100 mg/kg twice a day. Regorafenib was orally administered at 5 mg/kg every day.

### Real-time quantitative PCR

Total DNA was extracted from CRC cells using Animal Tissues/Cells Genomic DNA Extraction Kit (Solarbio Science and Technology, Beijing, China), according to the manufacturer’s instruction. Quantitative PCR was performed using Hieff^®^ qPCR SYBR Green Master Mix (Yeasen Biotech, Shanghai, China) and Step-One Plus Real-Time PCR Systems (Applied Biosystems, Inc., Foster City, CA, USA). A pair of BRAF mutated V600E primers were used in this study. The first primers were forward-5′-CCTAAACTCTTCATAATGCTTGCTC-3′ and reverse-5′-TCAGGGCCAAAAATTTAATCAG-3′ (Sangon Biotech, Shanghai, China). Beta-actin was used as an internal control.

### Western blotting

The following antibodies were used: PARP, phospho-STAT3 (Ser727 and Thr705), ERK1/2 and phospho-ERK1/2 (Thr202/Tyr204), PUMA, MDM2, NOXA and thymidine synthase (TS) (Cell Signaling Technology, Beverly, MA, USA), STAT3 and P53 (Santa Cruz Biotechnology, Santa Cruz, CA, USA); VEGFA and ALDH1a (Abcam, USA). Cells were collected and lysed using RIPA protein lysis buffer. Total protein was collected and quantified using the Bio-Rad Bradford reagent (Bio-Rad Laboratories, Hercules, CA, USA). Equal amounts of protein were separated in 10–15% SDS-PAGE gels using the XCell system (Invitrogen). The separated proteins were transferred to PVDF membranes (Millipore) using a transfer apparatus (Bio-Rad). After blocking with 10% (w/v) nonfat-milk in Tris-HCL buffered saline containing 0.1% tween20 (TBST), the blots were incubated with primary antibodies at 4°C overnight, and subsequently incubated with the corresponding fluorescent secondary antibodies, and the bands were visualized using Odyssey Infrared Imaging System (LI-COR Bioscience, Lincoln, NE, USA).

### Statistical analysis

Data are shown as average ± standard error (Mean ± SEM) and were analyzed by the Student’s *t*-test (two-tailed, unpaired or paired) using Prism GraphPad 5 software. Each cell experiment was repeated at least 3 times independently. A *p*-value of less than 0.05 was considered to be statistically significant.

## References

[1] Mayer RJ , Van Cutsem E , Falcone A , Yoshino T , Garcia-Carbonero R , Mizunuma N , Yamazaki K , Shimada Y , Tabernero J , Komatsu Y , Sobrero A , Boucher E , Peeters M , et al, and RECOURSE Study Group. Randomized trial of TAS-102 for refractory metastatic colorectal cancer. N Engl J Med. 2015; 372:1909–19. 10.1056/NEJMoa1414325. 25970050

[2] Arnold D , Prager GW , Quintela A , Stein A , Moreno Vera S , Mounedji N , Taieb J . Beyond second-line therapy in patients with metastatic colorectal cancer: a systematic review. Ann Oncol. 2018; 29:835–56. 10.1093/annonc/mdy038. 29452346 PMC5913602

[3] Ilson DH , Tabernero J , Prokharau A , Arkenau HT , Ghidini M , Fujitani K , Van Cutsem E , Thuss-Patience P , Beretta GD , Mansoor W , Zhavrid E , Alsina M , George B , et al. Efficacy and Safety of Trifluridine/Tipiracil Treatment in Patients With Metastatic Gastric Cancer Who Had Undergone Gastrectomy: Subgroup Analyses of a Randomized Clinical Trial. JAMA Oncol. 2020; 6:e193531. 10.1001/jamaoncol.2019.3531. 31600365 PMC6802061

[4] Shitara K , Doi T , Dvorkin M , Mansoor W , Arkenau HT , Prokharau A , Alsina M , Ghidini M , Faustino C , Gorbunova V , Zhavrid E , Nishikawa K , Hosokawa A , et al. Trifluridine/tipiracil versus placebo in patients with heavily pretreated metastatic gastric cancer (TAGS): a randomised, double-blind, placebo-controlled, phase 3 trial. Lancet Oncol. 2018; 19:1437–48. 10.1016/S1470-2045(18)30739-3. 30355453

[5] van der Velden DL , Opdam FL , Voest EE . TAS-102 for Treatment of Advanced Colorectal Cancers That Are No Longer Responding to Other Therapies. Clin Cancer Res. 2016; 22:2835–39. 10.1158/1078-0432.CCR-15-2783. 27126991

[6] Xu J , Kim TW , Shen L , Sriuranpong V , Pan H , Xu R , Guo W , Han SW , Liu T , Park YS , Shi C , Bai Y , Bi F , et al. Results of a Randomized, Double-Blind, Placebo-Controlled, Phase III Trial of Trifluridine/Tipiracil (TAS-102) Monotherapy in Asian Patients With Previously Treated Metastatic Colorectal Cancer: The TERRA Study. J Clin Oncol. 2018; 36:350–58. 10.1200/JCO.2017.74.3245. 29215955

[7] Kuboki Y , Nishina T , Shinozaki E , Yamazaki K , Shitara K , Okamoto W , Kajiwara T , Matsumoto T , Tsushima T , Mochizuki N , Nomura S , Doi T , Sato A , et al. TAS-102 plus bevacizumab for patients with metastatic colorectal cancer refractory to standard therapies (C-TASK FORCE): an investigator-initiated, open-label, single-arm, multicentre, phase 1/2 study. Lancet Oncol. 2017; 18:1172–81. 10.1016/S1470-2045(17)30425-4. 28760399

[8] Baba Y , Tamura T , Satoh Y , Gotou M , Sawada H , Ebara S , Shibuya K , Soeda J , Nakamura K . Panitumumab interaction with TAS-102 leads to combinational anticancer effects via blocking of EGFR-mediated tumor response to trifluridine. Mol Oncol. 2017; 11:1065–77. 10.1002/1878-0261.12074. 28486761 PMC5537908

[9] Suzuki N , Nakagawa F , Matsuoka K , Takechi T . Effect of a novel oral chemotherapeutic agent containing a combination of trifluridine, tipiracil and the novel triple angiokinase inhibitor nintedanib, on human colorectal cancer xenografts. Oncol Rep. 2016; 36:3123–30. 10.3892/or.2016.5208. 27805254 PMC5112602

[10] Suzuki N , Tsukihara H , Nakagawa F , Kobunai T , Takechi T . Synergistic anticancer activity of a novel oral chemotherapeutic agent containing trifluridine and tipiracil in combination with anti-PD-1 blockade in microsatellite stable-type murine colorectal cancer cells. Am J Cancer Res. 2017; 7:2032–40. 29119052 PMC5665850

[11] Li J , Qin S , Xu R , Yau TC , Ma B , Pan H , Xu J , Bai Y , Chi Y , Wang L , Yeh KH , Bi F , Cheng Y , et al. Regorafenib plus best supportive care versus placebo plus best supportive care in Asian patients with previously treated metastatic colorectal cancer (CONCUR): a randomised, double-blind, placebo-controlled, phase 3 trial. Lancet Oncol. 2015; 16:619–29. 10.1016/S1470-2045(15)70156-7. 25981818

[12] Bruix J , Qin S , Merle P , Granito A , Huang YH , Bodoky G , Pracht M , Yokosuka O , Rosmorduc O , Breder V , Gerolami R , Masi G , Ross PJ , et al, and RESORCE Investigators. Regorafenib for patients with hepatocellular carcinoma who progressed on sorafenib treatment (RESORCE): a randomised, double-blind, placebo-controlled, phase 3 trial. Lancet. 2017; 389:56–66. 10.1016/S0140-6736(16)32453-9. 27932229

[13] Burki TK . Progression-free survival with regorafenib in gastric cancer. Lancet Oncol. 2016; 17:e323. 10.1016/S1470-2045(16)30284-4. 27375105

[14] Tong M , Che N , Zhou L , Luk ST , Kau PW , Chai S , Ngan ES , Lo CM , Man K , Ding J , Lee TK , Ma S . Efficacy of annexin A3 blockade in sensitizing hepatocellular carcinoma to sorafenib and regorafenib. J Hepatol. 2018; 69:826–39. 10.1016/j.jhep.2018.05.034. 29885413

[15] Zhang WJ , Li Y , Wei MN , Chen Y , Qiu JG , Jiang QW , Yang Y , Zheng DW , Qin WM , Huang JR , Wang K , Zhang WJ , Wang YJ , et al. Synergistic antitumor activity of regorafenib and lapatinib in preclinical models of human colorectal cancer. Cancer Lett. 2017; 386:100–9. 10.1016/j.canlet.2016.11.011. 27864115

[16] Kim J , Ulu A , Wan D , Yang J , Hammock BD , Weiss RH . Addition of DHA Synergistically Enhances the Efficacy of Regorafenib for Kidney Cancer Therapy. Mol Cancer Ther. 2016; 15:890–98. 10.1158/1535-7163.MCT-15-0847. 26921392 PMC4873345

[17] Tsai AK , Khan AY , Worgo CE , Wang LL , Liang Y , Davila E . A Multikinase and DNA-PK Inhibitor Combination Immunomodulates Melanomas, Suppresses Tumor Progression, and Enhances Immunotherapies. Cancer Immunol Res. 2017; 5:790–803. 10.1158/2326-6066.CIR-17-0009. 28775208 PMC5626455

[18] Marks EI , Tan C , Zhang J , Zhou L , Yang Z , Scicchitano A , El-Deiry WS . Regorafenib with a fluoropyrimidine for metastatic colorectal cancer after progression on multiple 5-FU-containing combination therapies and regorafenib monotherapy. Cancer Biol Ther. 2015; 16:1710–19. 10.1080/15384047.2015.1113355. 26561209 PMC4847811

[19] Lytle NK , Barber AG , Reya T . Stem cell fate in cancer growth, progression and therapy resistance. Nat Rev Cancer. 2018; 18:669–80. 10.1038/s41568-018-0056-x. 30228301 PMC8388042

[20] Zhao Y , Dong Q , Li J , Zhang K , Qin J , Zhao J , Sun Q , Wang Z , Wartmann T , Jauch KW , Nelson PJ , Qin L , Bruns C . Targeting cancer stem cells and their niche: perspectives for future therapeutic targets and strategies. Semin Cancer Biol. 2018; 53:139–55. 10.1016/j.semcancer.2018.08.002. 30081228

[21] Suenaga M , Schirripa M , Cao S , Zhang W , Yang D , Dadduzio V , Salvatore L , Borelli B , Pietrantonio F , Ning Y , Okazaki S , Berger MD , Miyamoto Y , et al. Potential role of polymorphisms in the transporter genes ENT1 and MATE1/OCT2 in predicting TAS-102 efficacy and toxicity in patients with refractory metastatic colorectal cancer. Eur J Cancer. 2017; 86:197–206. 10.1016/j.ejca.2017.08.033. 28992563 PMC7497848

[22] Her Z , Yong KSM , Paramasivam K , Tan WWS , Chan XY , Tan SY , Liu M , Fan Y , Linn YC , Hui KM , Surana U , Chen Q . An improved pre-clinical patient-derived liquid xenograft mouse model for acute myeloid leukemia. J Hematol Oncol. 2017; 10:162. 10.1186/s13045-017-0532-x. 28985760 PMC5639594

[23] Takigawa H , Kitadai Y , Shinagawa K , Yuge R , Higashi Y , Tanaka S , Yasui W , Chayama K . Multikinase inhibitor regorafenib inhibits the growth and metastasis of colon cancer with abundant stroma. Cancer Sci. 2016; 107:601–8. 10.1111/cas.12907. 26865419 PMC5001714

[24] Väyrynen SA , Väyrynen JP , Klintrup K , Mäkelä J , Karttunen TJ , Tuomisto A , Mäkinen MJ . Clinical impact and network of determinants of tumour necrosis in colorectal cancer. Br J Cancer. 2016; 114:1334–42. 10.1038/bjc.2016.128. 27195424 PMC4984458

[25] Lee SY , Ju MK , Jeon HM , Jeong EK , Lee YJ , Kim CH , Park HG , Han SI , Kang HS . Regulation of Tumor Progression by Programmed Necrosis. Oxid Med Cell Longev. 2018; 2018:3537471. 10.1155/2018/3537471. 29636841 PMC5831895

[26] Karsch-Bluman A , Feiglin A , Arbib E , Stern T , Shoval H , Schwob O , Berger M , Benny O . Tissue necrosis and its role in cancer progression. Oncogene. 2019; 38:1920–35. 10.1038/s41388-018-0555-y. 30390074

[27] Patel AK , Barghout V , Yenikomshian MA , Germain G , Jacques P , Laliberté F , Duh MS . Real-World Adherence in Patients with Metastatic Colorectal Cancer Treated with Trifluridine plus Tipiracil or Regorafenib. Oncologist. 2020; 25:e75–84. 10.1634/theoncologist.2019-0240. 31591140 PMC6964129

[28] Rycaj K , Tang DG . Cell-of-Origin of Cancer versus Cancer Stem Cells: Assays and Interpretations. Cancer Res. 2015; 75:4003–11. 10.1158/0008-5472.CAN-15-0798. 26292361 PMC4756645

[29] Kaushik V , Yakisich JS , Way LF , Azad N , Iyer AKV . Chemoresistance of cancer floating cells is independent of their ability to form 3D structures: Implications for anticancer drug screening. J Cell Physiol. 2019; 234:4445–53. 10.1002/jcp.27239. 30191978

[30] Shaheen S , Ahmed M , Lorenzi F , Nateri AS . Spheroid-Formation (Colonosphere) Assay for in Vitro Assessment and Expansion of Stem Cells in Colon Cancer. Stem Cell Rev Rep. 2016; 12:492–99. 10.1007/s12015-016-9664-6. 27207017 PMC4919387

[31] Liu L , Cao Y , Chen C , Zhang X , McNabola A , Wilkie D , Wilhelm S , Lynch M , Carter C . Sorafenib blocks the RAF/MEK/ERK pathway, inhibits tumor angiogenesis, and induces tumor cell apoptosis in hepatocellular carcinoma model PLC/PRF/5. Cancer Res. 2006; 66:11851–58. 10.1158/0008-5472.CAN-06-1377. 17178882

[32] Zhang Q , Lu S , Li T , Yu L , Zhang Y , Zeng H , Qian X , Bi J , Lin Y . ACE2 inhibits breast cancer angiogenesis via suppressing the VEGFa/VEGFR2/ERK pathway. J Exp Clin Cancer Res. 2019; 38:173. 10.1186/s13046-019-1156-5. 31023337 PMC6482513

[33] Wang M , Zhang C , Zheng Q , Ma Z , Qi M , Di G , Ling S , Xu H , Qi B , Yao C , Xia H , Jiang X . RhoJ facilitates angiogenesis in glioblastoma via JNK/VEGFR2 mediated activation of PAK and ERK signaling pathways. Int J Biol Sci. 2022; 18:942–55. 10.7150/ijbs.65653. 35173528 PMC8771846

[34] Matsuoka K , Iimori M , Niimi S , Tsukihara H , Watanabe S , Kiyonari S , Kiniwa M , Ando K , Tokunaga E , Saeki H , Oki E , Maehara Y , Kitao H . Trifluridine Induces p53-Dependent Sustained G2 Phase Arrest with Its Massive Misincorporation into DNA and Few DNA Strand Breaks. Mol Cancer Ther. 2015; 14:1004–13. 10.1158/1535-7163.MCT-14-0236. 25700705

[35] Modest DP , Pant S , Sartore-Bianchi A . Treatment sequencing in metastatic colorectal cancer. Eur J Cancer. 2019; 109:70–83. 10.1016/j.ejca.2018.12.019. 30690295

[36] Martins-Neves SR , Cleton-Jansen AM , Gomes CMF . Therapy-induced enrichment of cancer stem-like cells in solid human tumors: Where do we stand? Pharmacol Res. 2018; 137:193–204. 10.1016/j.phrs.2018.10.011. 30316903

[37] Tsunekuni K , Konno M , Haraguchi N , Koseki J , Asai A , Matsuoka K , Kobunai T , Takechi T , Doki Y , Mori M , Ishii H . CD44/CD133-Positive Colorectal Cancer Stem Cells are Sensitive to Trifluridine Exposure. Sci Rep. 2019; 9:14861. 10.1038/s41598-019-50968-6. 31619711 PMC6795793

[38] Cai MH , Xu XG , Yan SL , Sun Z , Ying Y , Wang BK , Tu YX . Regorafenib suppresses colon tumorigenesis and the generation of drug resistant cancer stem-like cells via modulation of miR-34a associated signaling. J Exp Clin Cancer Res. 2018; 37:151. 10.1186/s13046-018-0836-x. 30005681 PMC6045878

[39] Ciccone V , Terzuoli E , Donnini S , Giachetti A , Morbidelli L , Ziche M . Stemness marker ALDH1A1 promotes tumor angiogenesis via retinoic acid/HIF-1α/VEGF signalling in MCF-7 breast cancer cells. J Exp Clin Cancer Res. 2018; 37:311. 10.1186/s13046-018-0975-0. 30541574 PMC6291966

[40] Biswas AK , Han S , Tai Y , Ma W , Coker C , Quinn SA , Shakri AR , Zhong TJ , Scholze H , Lagos GG , Mela A , Manova-Todorova K , de Stanchina E , et al. Targeting S100A9-ALDH1A1-Retinoic Acid Signaling to Suppress Brain Relapse in EGFR-Mutant Lung Cancer. Cancer Discov. 2022; 12:1002–21. 10.1158/2159-8290.CD-21-0910. 35078784 PMC8983473

[41] Nacarelli T , Fukumoto T , Zundell JA , Fatkhutdinov N , Jean S , Cadungog MG , Borowsky ME , Zhang R . NAMPT Inhibition Suppresses Cancer Stem-like Cells Associated with Therapy-Induced Senescence in Ovarian Cancer. Cancer Res. 2020; 80:890–900. 10.1158/0008-5472.CAN-19-2830. 31857293 PMC7024650

[42] Suenaga M , Schirripa M , Cao S , Zhang W , Yang D , Murgioni S , Rossini D , Marmorino F , Mennitto A , Ning Y , Okazaki S , Berger MD , Miyamoto Y , et al. Genetic variants of DNA repair-related genes predict efficacy of TAS-102 in patients with refractory metastatic colorectal cancer. Ann Oncol. 2017; 28:1015–22. 10.1093/annonc/mdx035. 28453695 PMC5834058

[43] Rodríguez-Hernández MA , Chapresto-Garzón R , Cadenas M , Navarro-Villarán E , Negrete M , Gómez-Bravo MA , Victor VM , Padillo FJ , Muntané J . Differential effectiveness of tyrosine kinase inhibitors in 2D/3D culture according to cell differentiation, p53 status and mitochondrial respiration in liver cancer cells. Cell Death Dis. 2020; 11:339. 10.1038/s41419-020-2558-1. 32382022 PMC7206079

[44] Ricciuti B , Foglietta J , Bianconi V , Sahebkar A , Pirro M . Enzymes involved in tumor-driven angiogenesis: A valuable target for anticancer therapy. Semin Cancer Biol. 2019; 56:87–99. 10.1016/j.semcancer.2017.11.005. 29128510

[45] Tai WT , Chu PY , Shiau CW , Chen YL , Li YS , Hung MH , Chen LJ , Chen PL , Su JC , Lin PY , Yu HC , Chen KF . STAT3 mediates regorafenib-induced apoptosis in hepatocellular carcinoma. Clin Cancer Res. 2014; 20:5768–76. 10.1158/1078-0432.CCR-14-0725. 25248379

[46] Zhuo J , Lu D , Lin Z , Yang X , Yang M , Wang J , Tao Y , Wen X , Li H , Lian Z , Cen B , Dong S , Wei X , et al. The distinct responsiveness of cytokeratin 19-positive hepatocellular carcinoma to regorafenib. Cell Death Dis. 2021; 12:1084. 10.1038/s41419-021-04320-4. 34785656 PMC8595883

[47] Yang C , Song J , Hwang S , Choi J , Song G , Lim W . Apigenin enhances apoptosis induction by 5-fluorouracil through regulation of thymidylate synthase in colorectal cancer cells. Redox Biol. 2021; 47:102144. 10.1016/j.redox.2021.102144. 34562873 PMC8476449

[48] Arnaoutova I , George J , Kleinman HK , Benton G . The endothelial cell tube formation assay on basement membrane turns 20: state of the science and the art. Angiogenesis. 2009; 12:267–74. 10.1007/s10456-009-9146-4. 19399631

[49] Vartanian RK , Weidner N . Endothelial cell proliferation in prostatic carcinoma and prostatic hyperplasia: correlation with Gleason’s score, microvessel density, and epithelial cell proliferation. Lab Invest. 1995; 73:844–50. 8558846

[50] Zhang J , Zhou L , Zhao S , Dicker DT , El-Deiry WS . The CDK4/6 inhibitor palbociclib synergizes with irinotecan to promote colorectal cancer cell death under hypoxia. Cell Cycle. 2017; 16:1193–200. 10.1080/15384101.2017.1320005. 28486050 PMC5499912

[51] Chou TC . Drug combination studies and their synergy quantification using the Chou-Talalay method. Cancer Res. 2010; 70:440–46. 10.1158/0008-5472.CAN-09-1947. 20068163

